# The impact of electron streaming in head and neck cancer patients treated with ultra-hypofractionated MR-guided radiotherapy

**DOI:** 10.1016/j.phro.2026.101117

**Published:** 2026-11-02

**Authors:** Sabine M.L. Linden, Mischa de Ridder, Bas W. Raaymakers, Eric Brand, Marielle E.P. Philippens, Cornelis P.J. Raaijmakers

**Affiliations:** Department of Radiotherapy, University Medical Center Utrecht, Heidelberglaan 100, 3584, CX, Utrecht, the Netherlands

**Keywords:** Early-stage glottic cancer, Magnetic Resonance-guided radiotherapy, Ultra-hypofractionation, Electron streaming effect

## Abstract

This study aims to quantify the influence of the electron streaming effect for MR-guided treatment regimens in head and neck cancer. Treatment plans of ten patients treated with an ultra-hypofractionated regimen on a 1.5 T MR-Linac were analyzed. Unwanted dose deposition several centimeters cranial and caudal from the high-dose region was clearly present. However, the out-of-field D_max_ in the mucosa surrounding the air-filled cavities and the skin remained well below the dose range associated with toxicity. These findings do not preclude future clinical trials investigating MR-guided ultra-hypofractionated treatment regimens for head and neck cancer.

## Highlights

• The electron streaming effect was clearly observed in head and neck cancer patients.
• The dosimetric effects observed are unlikely to be clinically relevant.
• These findings do not preclude magnetic resonance-guided radiotherapy for head and neck cancer.

## Graphical abstract

**Figure:**
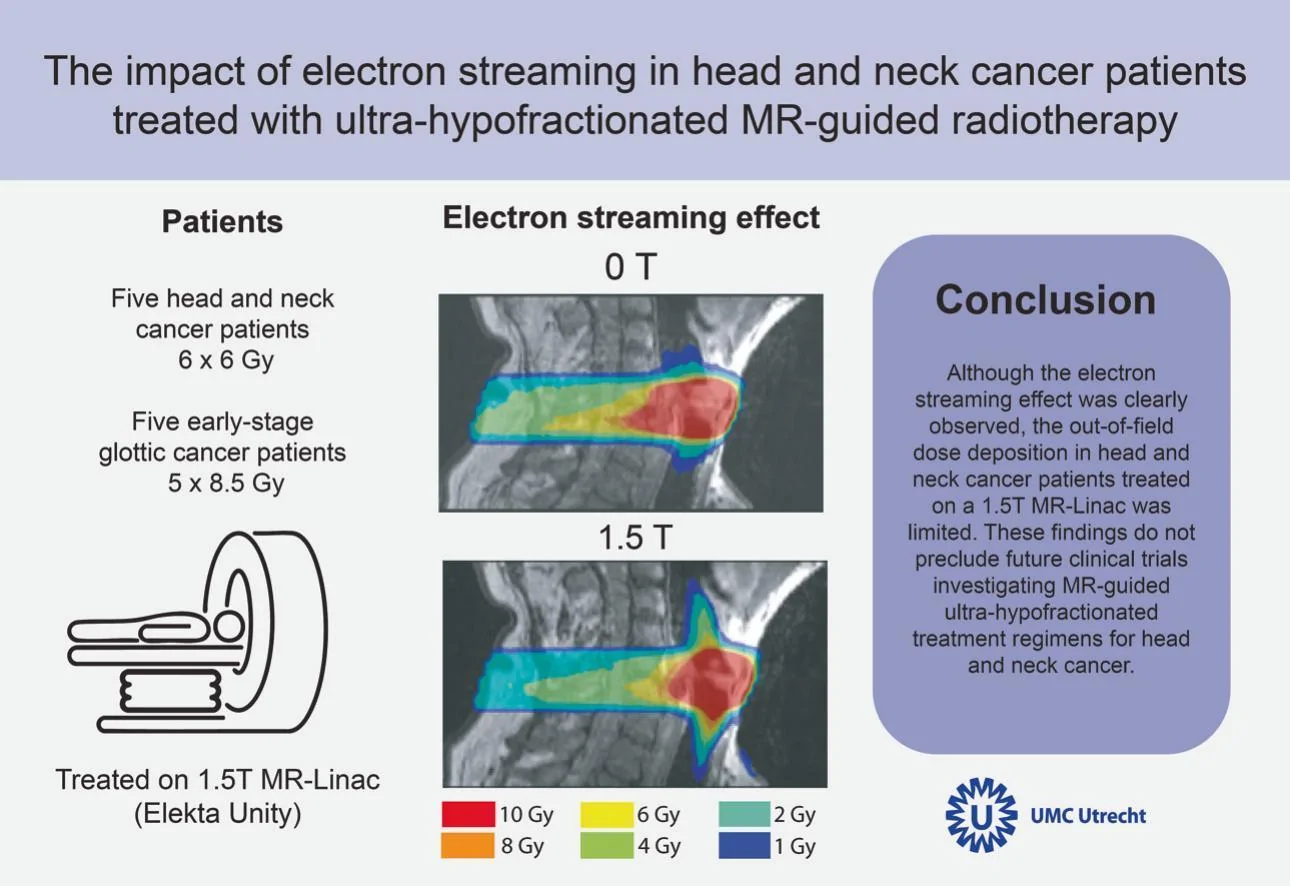

## 1 Introduction

The use of MR-guided radiotherapy for head and neck cancer patients is increasing [1], [2], [3]. The integration of soft-tissue imaging during treatment improves visualization of the target volume and the organs at risk, facilitating daily adaptive treatments [4]. In addition, motion management allows for tumor gating to account for intra-fraction motion [5], [6]. These features make treatments more precise and accurate, potentially decreasing radiation toxicity and enabling ultra-hypofractionated treatment regimens.

MR-guided radiotherapy can lead to dose deposition effects related to the magnetic field, notably the electron streaming effect (ESE) and the electron return effect (ERE) [7]. These effects arise from the influence of the Lorentz force on secondary electrons [7]. Unobstructed, the electrons can travel considerable distances through the air, spiraling along the magnetic field lines [8]. In the head and neck area, the tumor is generally close to air-tissue interfaces, including the trachea, pharynx, oral/nasal cavities, and the skin. Consequently, unintended dose deposition outside the irradiated field may be more pronounced than for treatment sites lacking substantial tissue-air interfaces. Given the high fractional doses delivered in ultra-hypofractionated radiotherapy, understanding the extent of this effect is important. Current treatment planning systems incorporate magnetic field effects during optimization. However, the associated dose deposition in the mucosa surrounding the air-filled cavities is generally not evaluated as part of the treatment planning process and has not been previously described.

Despite growing awareness of these effects, most existing studies have focused on other anatomical sites [9], [10]. Plan robustness and an assessment of the ERE in the high-dose region were investigated for head and neck in two studies [11], [12]. The impact of the magnetic field on plan quality and ERE-induced dose increase was evaluated for 10 patients with hypopharyngeal carcinoma, who were treated with conventionally fractionated radiotherapy [12]. These studies did not report on the dose deposition outside of the high-dose target region, caused by the electron streaming effect.

At UMC Utrecht, MR-guided radiotherapy is currently used for two patient groups in the head and neck region: patients treated with a 6 × 6 Gy palliative regimen and early-stage glottic cancer patients treated with a 5 × 8.5 Gy regimen. This study aimed to quantify the impact of the electron streaming effect outside the high-dose target region for ultra-hypofractionated treatment regimens in head and neck cancer.

## 2 Methods

### 2.1 Patient selection

Head and neck cancer patients treated with a 6 × 6 Gy palliative regimen, and early-stage glottic cancer patients treated with a 5 × 8.5 Gy regimen (ARIA-study: NL-OMON57519*)* were selected for this study. All patients completed treatment on a 1.5 T MR-Linac (Elekta Unity) at University Medical Center Utrecht. Patients were included in the UMC Utrecht Umbrella Protocol Accelerator Imaging & Oncology (23 U-0036) accelerator study.

### 2.2 Imaging and treatment planning

Either MRI or CT was used for the pre-treatment delineation and treatment plan optimization. Clinically delivered treatment plans with a 1.5 T magnetic field were generated in Monaco (version 6.2.1). If the air-filled cavities were not delineated sufficiently for this analysis, this delineation was adjusted, and the treatment plan was recalculated. To evaluate the effect of the magnetic field, corresponding 0 T treatment plans were optimized in the research environment for conventional treatment planning in Monaco (version 6.00.01). The settings for the reference plan optimization were matched with the MR-Linac settings as closely as possible, including beam arrangement, isocenter position, grid size, optimization parameters, and the IMRT delivery technique.

### 2.3 Delineations outside the high-dose region

In order to focus on the low dose region where the ESE is expected, a high-dose region was defined as the total volume spanning the cranial and caudal extent of the 10 Gy isodose line. The mucosa surrounding the air-filled cavities was delineated with a thickness of 5 mm and subdivided into consecutive 10 mm segments, cranial and caudal to the high-dose region. For the skin, two structures with a 5 mm thickness were delineated: one cranial and one caudal to the high-dose region (Fig. S1). For both mucosal and skin structures, the D_max_ (D_0.03cc_) was evaluated to quantify the ESE.

## 3 Results

Five head and neck cancer patients treated with a 6 × 6 Gy palliative regimen were included: one patient with parotid gland cancer, one with laryngeal cancer, one with a tumor at the tongue, one with oropharyngeal cancer, and one with a hypopharyngeal tumor. Furthermore, five patients treated for early-stage glottic cancer were included. In all patients, the ESE was clearly visible in the air-filled cavities and/or on the skin up to several centimeters in cranial and caudal direction (Fig. 1, Table S2).

**Fig. 1:**
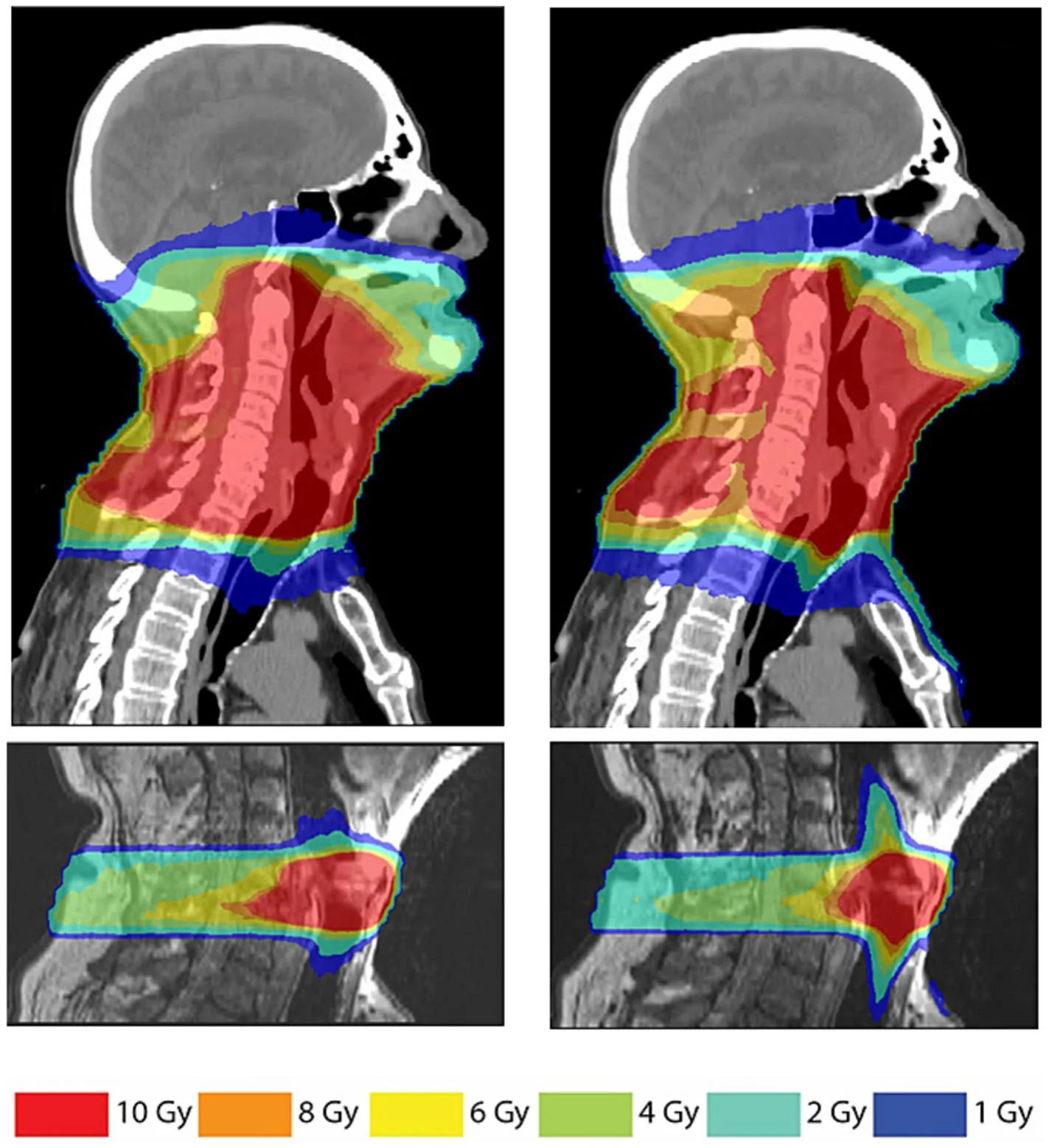
Dose distribution of a plan without magnetic field (left) and a plan with magnetic field (right) for a head and neck cancer patient treated with 6 × 6 Gy (upper) and an early-stage glottic cancer patient treated with 5 × 8.5 Gy (lower). The red color indicates a dose of >10 Gy for the total treatment plan. (For interpretation of the references to color in this figure legend, the reader is referred to the web version of this article.)

The magnitude of the ESE differed per patient and the maximum dose increase was 2.3 Gy (Fig. 2). The dose difference (D_max_ 1.5 T minus D_max_ 0 T) in the mucosa surrounding the air-filled cavities was positive for all patients at distances 10–60 mm, caudal to the high-dose region. Cranial to the high-dose region, the difference was positive in 70% of the cases. For the remaining 30%, the differences were within 0.3 Gy. At distances within 10 mm from the high-dose region, no consistent increase in dose values due to the ESE was observed (Table S3, Table S4).

**Fig. 2:**
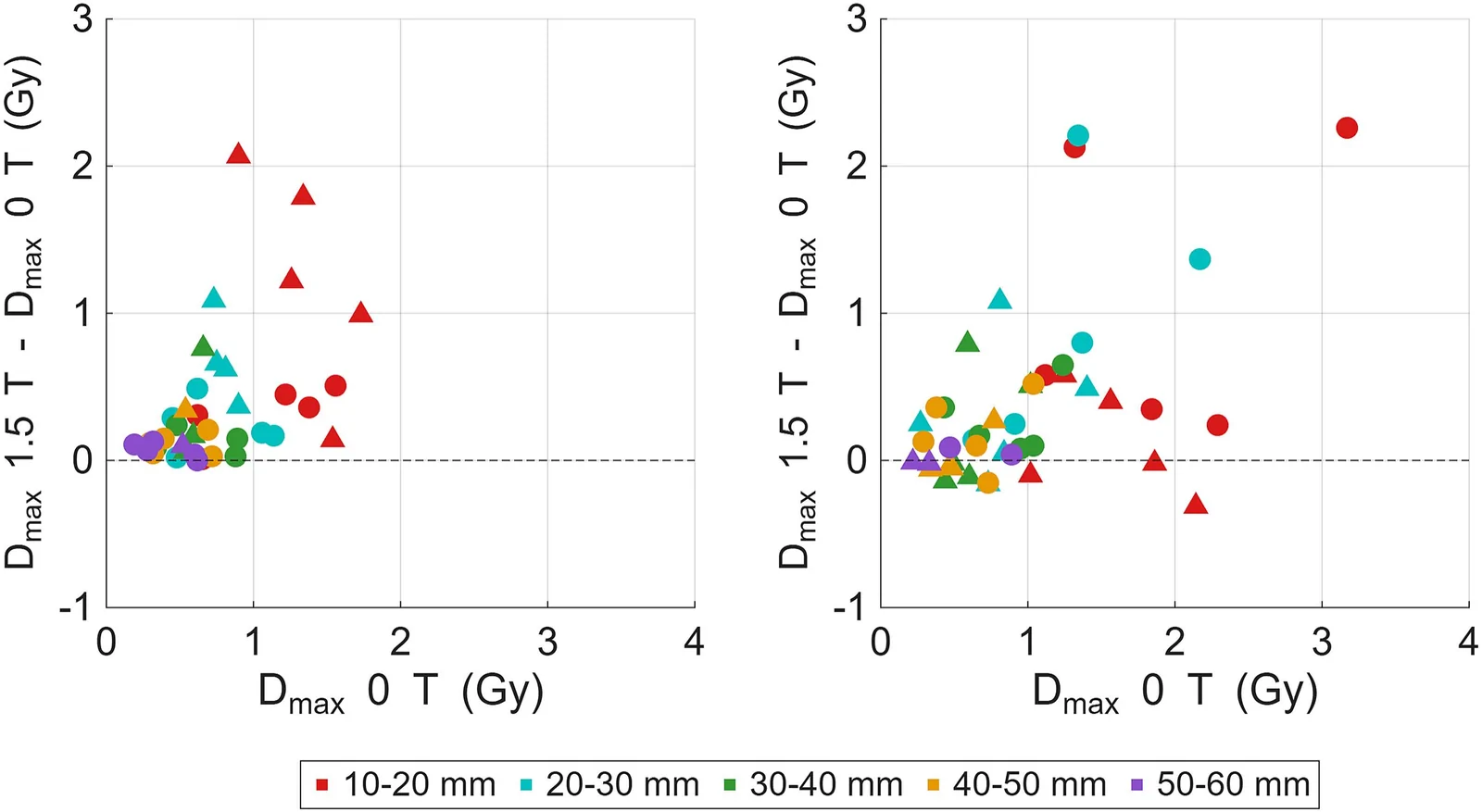
Maximum dose values in the 5 mm thick mucosa surrounding the air-filled cavities caudal to the high dose region (left) and cranial to the high dose region (right) for the total treatment plan. Colors indicate the distance to the high dose region (legend). Circles indicate the head and neck cancer patients treated with 6 × 6 Gy. Triangles indicate the early-stage glottic cancer patients treated with 5 × 8.5 Gy.

The D_max_ to the skin for patients treated with 6 × 6 Gy was higher for the plan with a magnetic field, compared to the plan without a magnetic field for 4/5 patients (Fig. S5). The maximal difference (D_max_ 1.5 T minus D_max_ 0 T) amounted to 3.6 Gy (caudal to the high-dose region). Visual assessment of the dose distributions indicated that the elevated D_max_ values caudal to the high-dose region in patient 2, 4 and 5 were caused by the ESE (Table S2). The average maximum skin dose for the plans with a magnetic field was 15% (5.3 Gy) of the total dose for these patients.

The D_max_ to the skin for patients treated with 5 × 8.5 Gy was not consistently higher for the plan with a magnetic field compared to the plan without magnetic field (Fig. S5). Visual assessment of the dose distributions indicated that the differences were not attributable to the ESE (Table S2). The average maximum skin dose outside the high-dose region for the plans with a magnetic field was 7% (2.8 Gy) for these patients.

## 4 Discussion

This study quantified the electron streaming effect in head and neck cancer patients treated on a 1.5 T MR-Linac. The ESE was clearly observed and caused a maximum increase of 2.3 Gy (D_max_) to the mucosa surrounding the air-filled cavities at a distance more than 10 mm outside the high-dose region. The maximal absolute dose on the mucosa outside the high-dose target region was 5.5 Gy (EQD2 = 4.3 Gy, using α/β = 3), remaining well below the dose range associated with mucositis [13], [14]. These dose values were observed at a distance of 10–60 mm outside the high-dose region. At a distance within 10 mm outside the high-dose region, no consistent increase was found. Although the ESE is present in this region, its contribution is outweighed by differences between the treatment plans that occur since the optimization algorithm attempts to compensate for differences due to these effects.

In the skin, a more pronounced effect was observed in the patients treated with a palliative treatment schedule compared to the early-stage glottic cancer patients. The maximum absolute dose difference of 7 Gy (EQD2 = 5.8 Gy, α/β = 3) was substantially lower than the dose range typically associated with the development of acute or late normal tissue toxicity for the skin, which generally starts at around 20 Gy [15].

The findings on increased skin dose for plans with a magnetic field are consistent with a study for esophageal cancer treatment [9] that reported a maximum skin dose of 15% of the prescription dose, on average. This is comparable to the skin dose we found for the 6 × 6 Gy patient group. In contrast, no consistent increase in skin dose was observed for the early-stage glottic cancer patients, likely due to the small high-dose target region and a less oblique entrance surface. The average maximum skin dose was 7% of the prescribed dose in this patient group. Differences in patient anatomy largely influence the magnitude of the ESE. Electrons in air can spiral around the magnetic field lines and travel over large distances. In the absence of intervening tissue along their trajectories, they may deposit their energy in tissue located at several centimeters from where they originated. ESE-related dose deposition outside the high-dose target region was limited and can be considered clinically negligible.

Our study was conducted on treatment plans that were delivered on a 1.5 T MR-Linac, providing a realistic assessment of the ESE during ultra-hypofractionated MR-guided radiotherapy. As the treatment planning system incorporates the magnetic field in generating a treatment plan, the treatment plan needed to be reoptimized in conventional treatment planning software. Re-optimization causes treatment plan differences, other than the magnetic field effects. Although the treatment plans with and without the magnetic field were mimicked as closely as possible during optimization, minor differences were unavoidable. The MR-Linac uses a 7 MV beam, whereas the conventional linac uses a 6 MV beam. This limited energy difference is expected to minimally affect the energy of the electrons in air and therefore on the assessment of the ESE. Another limitation is that the scan length of the MR scans for some early-stage glottic cancer patients was too small to encompass all predefined evaluation regions. This may have led to an underestimation of the ESE at larger distances. However, given the consistently low dose values observed, this is unlikely to affect the overall conclusion.

When reviewing MR-Linac treatment plans for the head and neck region, clinicians and medical physicists should be aware of the electron streaming effect. Our findings indicate, however, that the resulting dosimetric effects are not clinically relevant for these patient groups and do not preclude future clinical trials investigating MR-guided ultra-hypofractionated treatment regimens for head and neck cancer.

## CRediT authorship contribution statement

**Sabine M.L. Linden:** Writing – original draft, Visualization, Project administration, Methodology, Investigation, Formal analysis, Data curation, Conceptualization. **Mischa de Ridder:** Writing – review & editing, Visualization, Supervision, Methodology, Funding acquisition, Data curation, Conceptualization. **Bas W. Raaymakers:** Writing – review & editing. **Eric Brand:** Writing – review & editing, Supervision, Formal analysis. **Marielle E.P. Philippens:** Writing – review & editing, Visualization, Supervision, Methodology, Conceptualization. **Cornelis P.J. Raaijmakers:** Writing – review & editing, Visualization, Supervision, Methodology, Conceptualization.

## Ethics declaration

The authors have NOT obtained informed consent from participants or their legal representatives. Individual informed consent was not obtained specifically for this study. Patients who were alive at the time of the study had previously provided consent for the use of their clinical data for research purposes, including future studies. For patients who had died prior to the study, individual consent could no longer be obtained.

This study was performed in compliance with relevant laws, regulatory frameworks and guidelines where the research took place. Ethics committee approval was not required under relevant laws and institutional guidelines. This study falls under the UMC Utrecht Umbrella Protocol Accelerator Imaging & Oncology (23 U-0036). This does not fall under the scope of the Dutch Medical Research Involving Human Subjects Act (WMO) and therefore does not require approval from an accredited MREC in the Netherlands. For this study, all mandatory condition to conduct the study at the UMC Utrecht are met.

## Funding statement

This research was funded by UMC Utrecht.

## Declaration of competing interest

The authors declare that they have no known competing financial interests or personal relationships that could have appeared to influence the work reported in this paper.

## Data Availability

The data are not publicly available due to patient privacy, but are available from the corresponding author on reasonable request and with permission from UMC Utrecht.
