# Supplementary material for: The impact of electron streaming in head and neck cancer patients treated with ultra-hypofractionated MR-guided radiotherapy
Source: Phys Imaging Radiat Oncol. 2026 Nov 2;42:101117. doi: 10.1016/j.phro.2026.101117 (PMC13761243; doi:10.1016/j.phro.2026.101117)
Supplement: Supplementary file 1 [file mmc1.pdf]

Figure S1

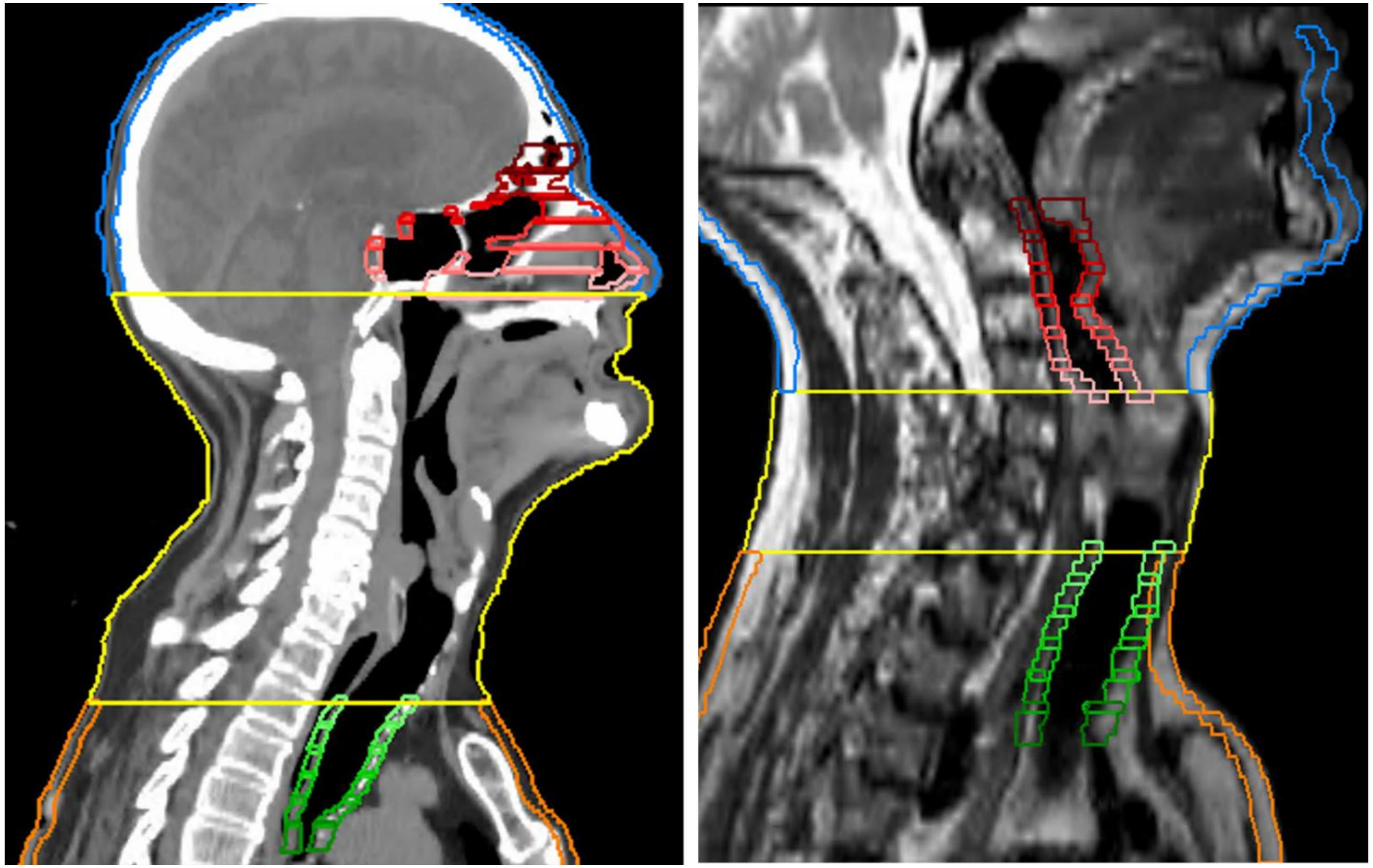

Consecutive 10-mm segments with 5-mm thickness of the mucosa caudal (green) and cranial (red) to the high-dose region (yellow). Skin structures with 5-mm thickness caudal (orange) and cranial (blue) to the high-dose region.

Table S2: Visualization of the CT/MRI, the delineations, the dose distribution with magnetic field, and the dose distribution without magnetic field.

<table>
<tr><th>Patient</th><th>Pre-treatment scan</th><th>Delineations</th><th>Dose distribution with magnetic field</th><th>Dose distribution without magnetic field</th></tr>
<tr><td></td><td></td><td>Yellow = high-dose volume
Orange = inferior skin
Blue = superior skin
Green = mucosa surrounding air-filled cavities, inferiorly.
Red = mucosa surrounding air-filled cavities, superiorly.</td><td>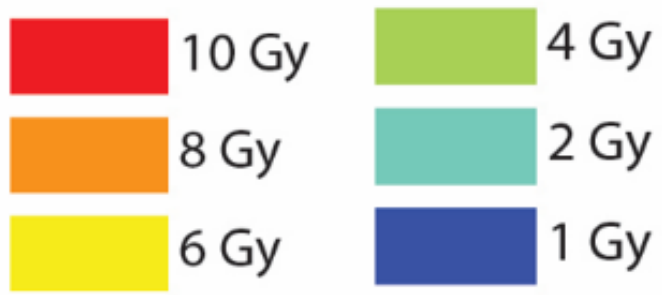
</td><td>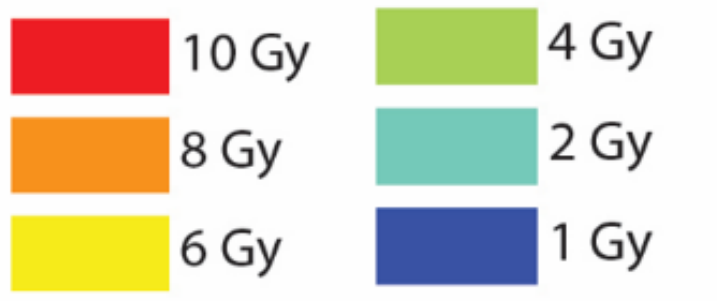
</td></tr>
<tr><td>Early-stage glottic cancer treated with 5 x 8.5 Gy (1)</td><td>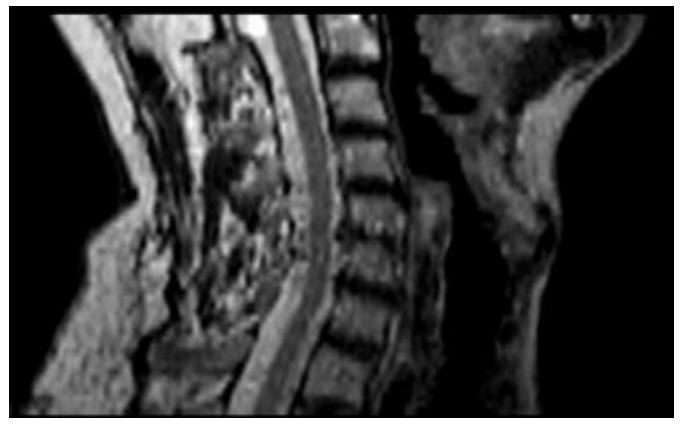</td><td>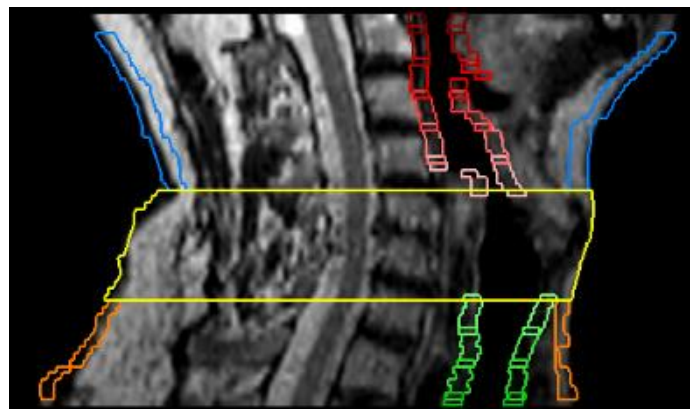</td><td>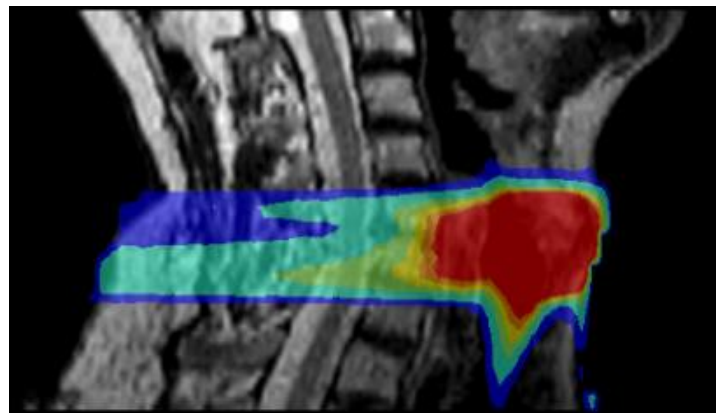</td><td>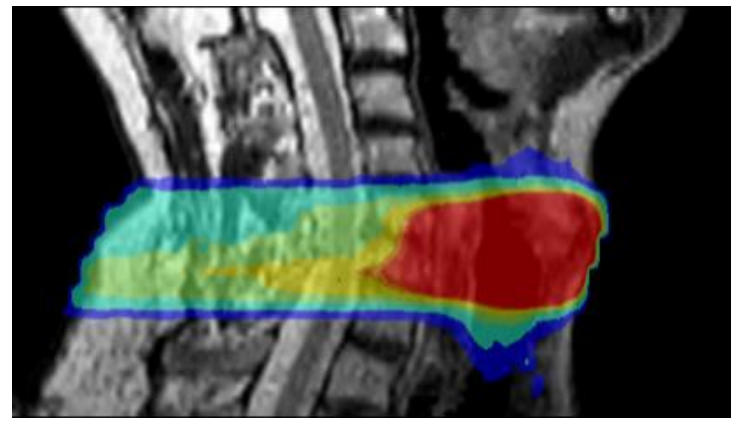</td></tr>
<tr><td>Early-stage glottic cancer treated with 5 x 8.5 Gy (2)</td><td>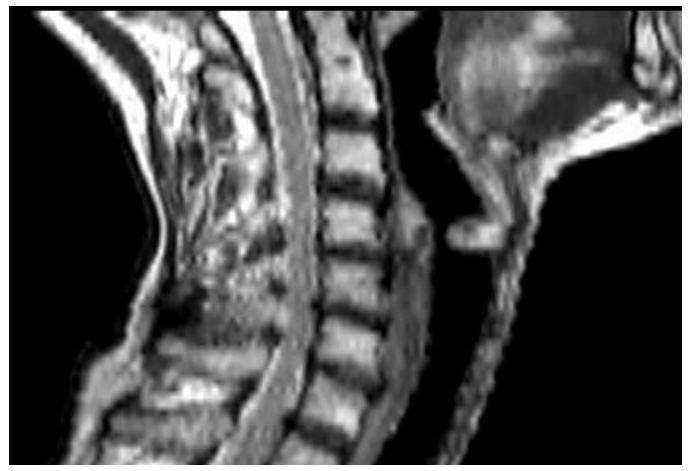</td><td>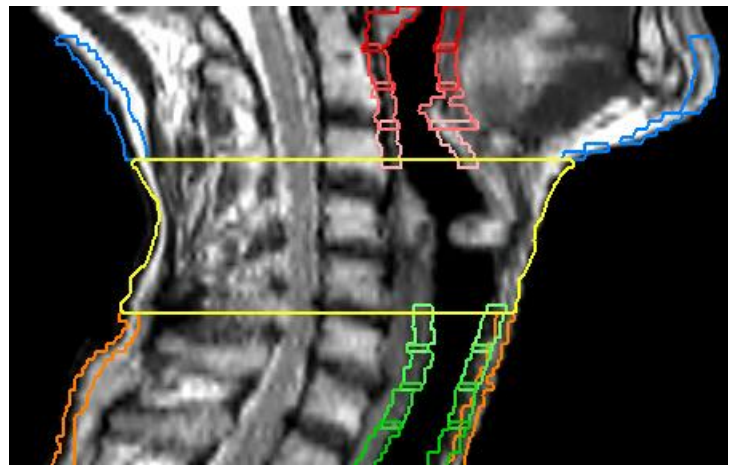</td><td>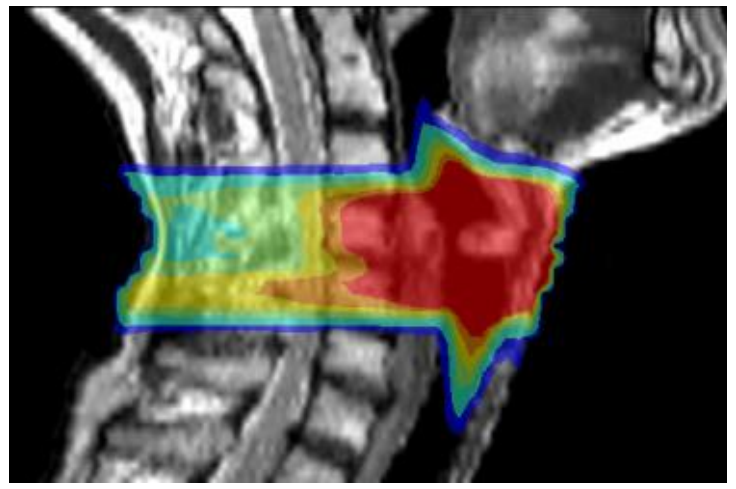</td><td>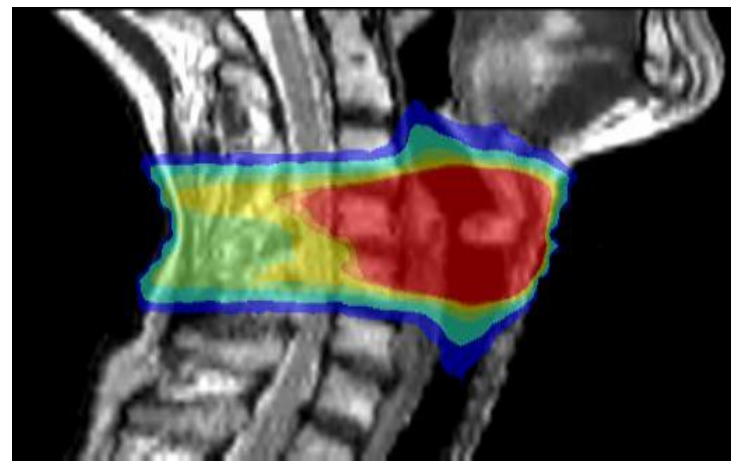</td></tr>
<tr><td>Early-stage glottic cancer treated with 5 x 8.5 Gy (3)</td><td>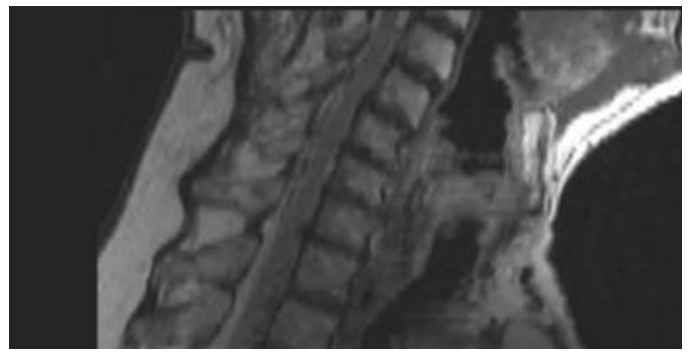</td><td>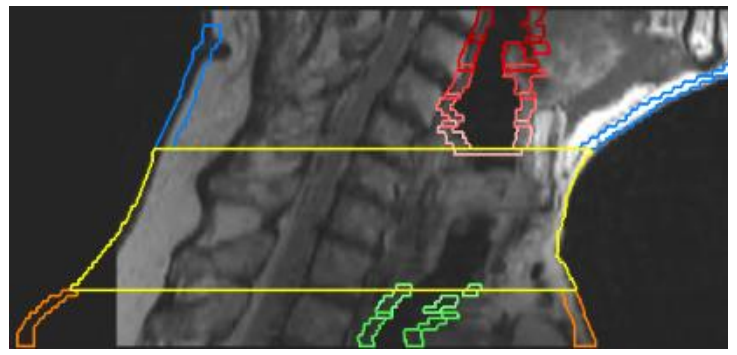</td><td>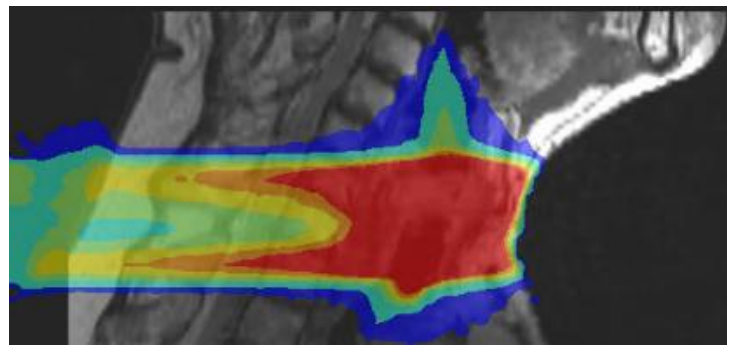</td><td>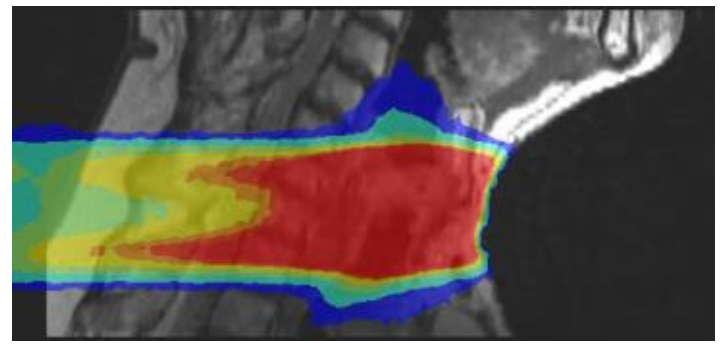</td></tr>
</table>

Early-stage glottic cancer treated with 5 x 8.5 Gy **(4)**

Early-stage glottic cancer treated with 5 x 8.5 Gy **(5)**

Head and neck cancer patient treated with 6 x 6 Gy **(1)**

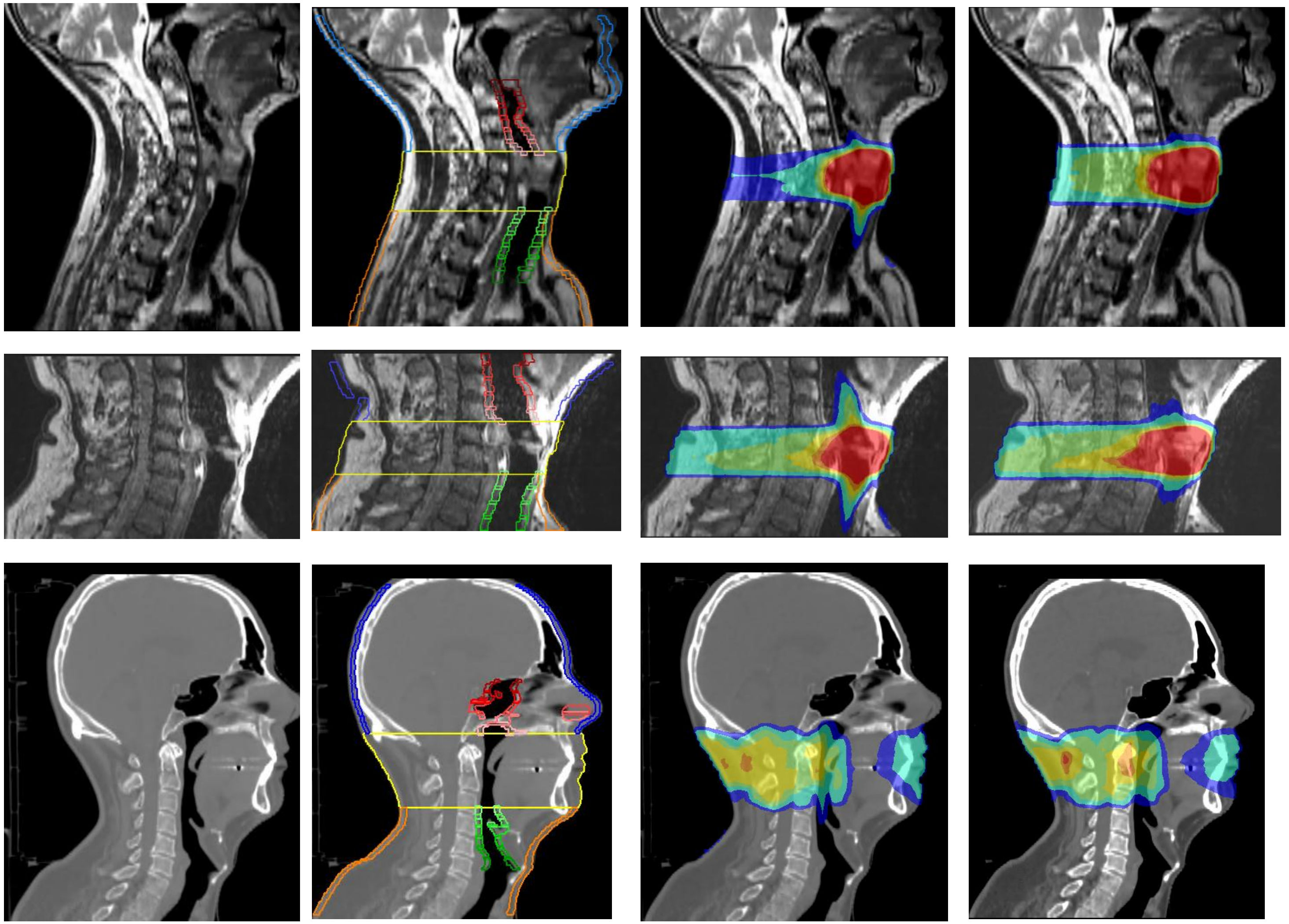

Head and neck cancer patient treated with 6 x 6 Gy **(2)**

Head and neck cancer patient treated with 6 x 6 Gy **(3)**

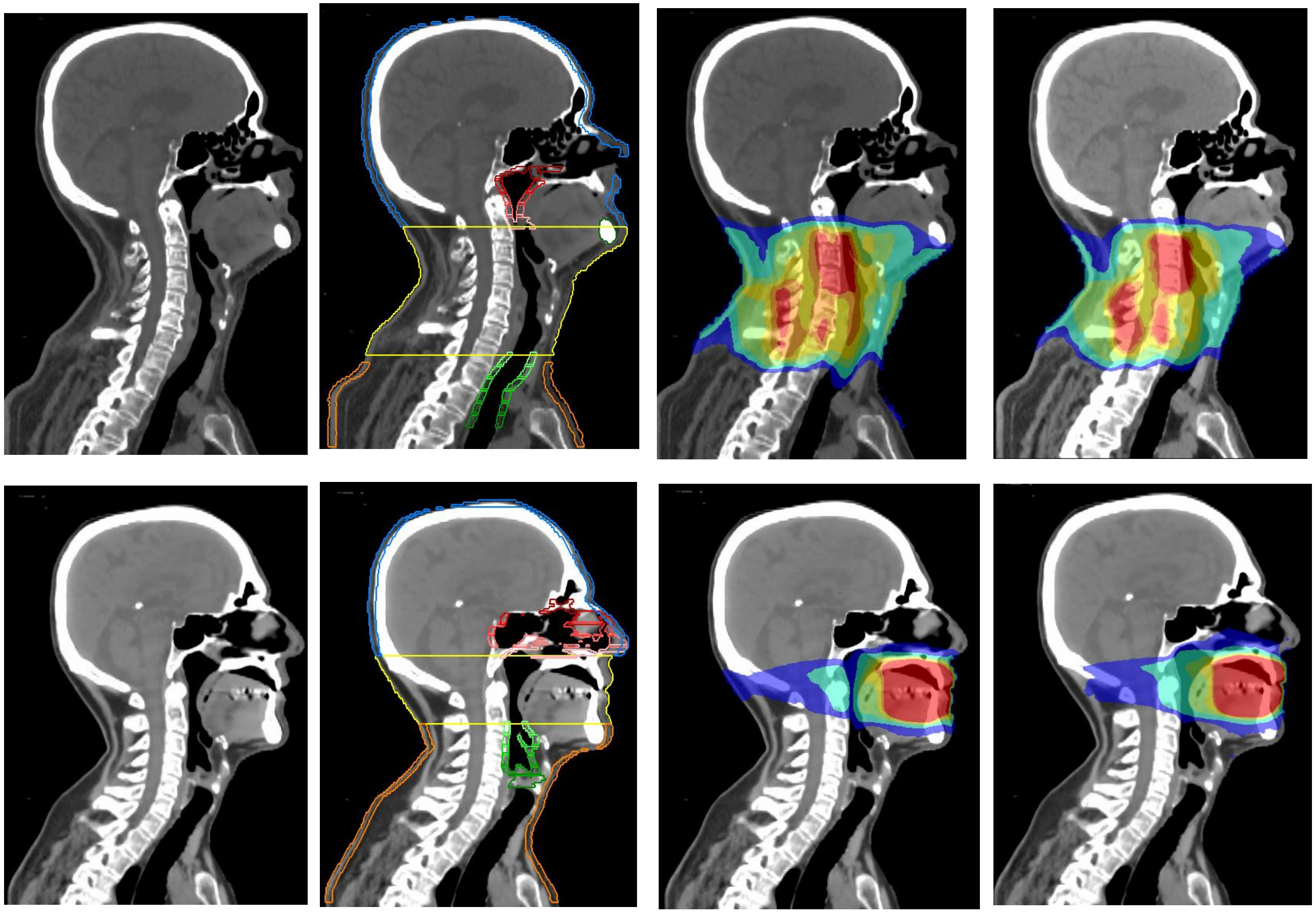

Head and neck cancer patient treated with 6 x 6 Gy **(4)**

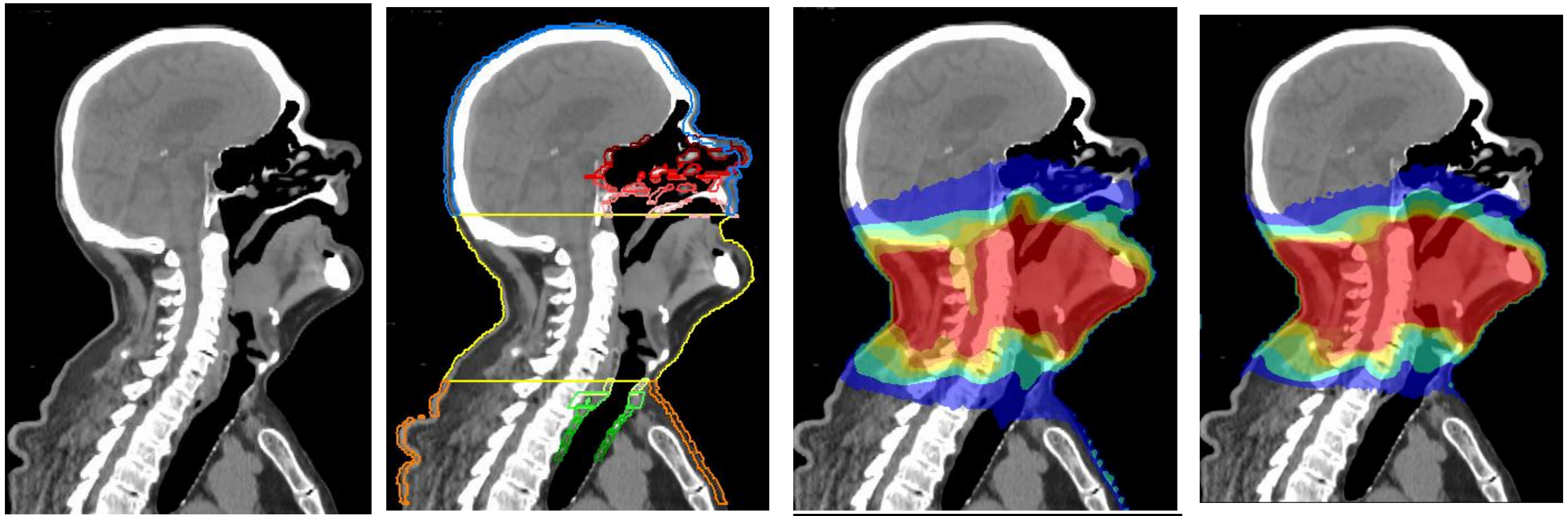

Head and neck cancer patient treated with 6 x 6 Gy **(5)**

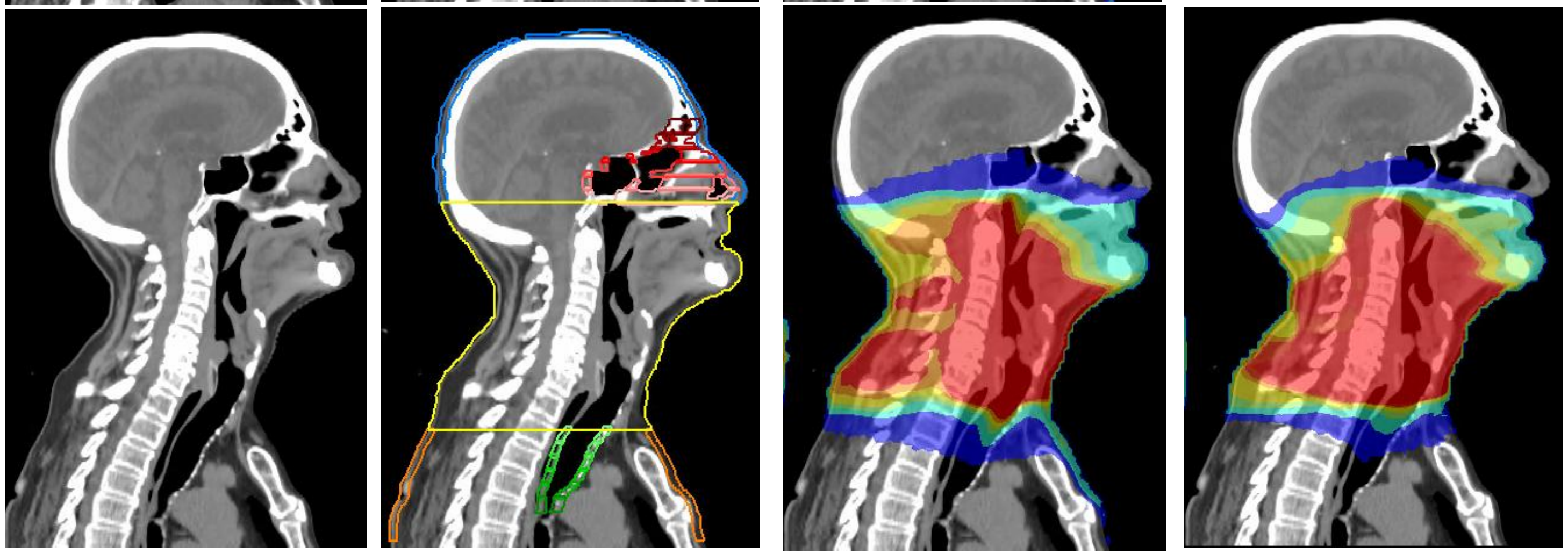

Table S3: Maximum dose values for early-stage glottic cancer patients in mucosal structures

Pt	Caudal 0-10 mm			Caudal 10-20 mm			Caudal 20-30 mm			Caudal 30-40 mm			Caudal 40-50 mm			Caudal 50-60 mm		
	1.5 T	0 T	Dif	1.5 T	0 T	Dif	1.5 T	0 T	Dif	1.5 T	0 T	Dif	1.5 T	0 T	Dif	1.5 T	0 T	Dif
1	8.3	9.8	-1.6	2.7	1.7	1.0	1.3	0.9	0.4	0.6	0.5	0.1						
2	6.0	4.9	1.0	2.5	1.3	1.2	1.4	0.8	0.6	0.6	0.6	0.1						
3	6.9	5.7	1.3	1.7	1.5	0.1												
4	6.2	3.8	2.4	3.0	0.9	2.1	1.8	0.7	1.1	1.4	0.7	0.8	0.9	0.5	0.3	0.6	0.5	0.1
5	4.8	5.1	-0.4	3.1	1.3	1.8	1.4	0.8	0.7	0.8	0.6	0.2						
Avg	6.4	5.9	0.5	2.6	1.4	1.2	1.5	0.8	0.7	0.9	0.6	0.3						
SD	1.2	2.1	1.4	0.5	0.3	0.7	0.2	0.1	0.3	0.3	0.1	0.3						
**Pt**	**Cranial 0-10 mm**			**Cranial 10-20 mm**			**Cranial 20-30 mm**			**Cranial 30-40 mm**			**Cranial 40-50 mm**			**Cranial 50-60 mm**		
	1.5 T	0 T	Dif	1.5 T	0 T	Dif	1.5 T	0 T	Dif	1.5 T	0 T	Dif	1.5 T	0 T	Dif	1.5 T	0 T	Dif
1	13.0	12.6	0.4	0.9	1.0	-0.1	0.5	0.3	0.3	0.3	0.4	-0.1	0.3	0.3	-0.1	0.2	0.2	0.0
2	8.2	6.9	1.2	2.0	1.6	0.4	0.6	0.7	-0.2	0.5	0.6	-0.1						
3	13.1	17.3	-4.2	1.8	2.1	-0.3	1.9	1.4	0.5	1.5	1.0	0.5	1.0	0.8	0.3			
4	18.6	24.9	-6.3	1.8	1.9	0.0	0.9	0.8	0.1	0.5	0.5	0.0	0.4	0.5	0.0	0.3	0.3	0.0
5	10.8	15.2	-4.4	1.8	1.3	0.6	1.9	0.8	1.1	1.4	0.6	0.8	0.4	0.5	-0.1			
Avg	12.7	15.4	-2.7	1.7	1.6	0.1	1.2	0.8	0.3	0.8	0.6	0.2	0.5	0.5	0.0	0.3	0.3	0.0
SD	3.4	5.9	2.9	0.4	0.4	0.3	0.6	0.4	0.4	0.5	0.2	0.4	0.3	0.2	0.1	0.1	0.1	0.0

**All values are presented in Gray (Gy). Pt=patient. Dif = difference 1.5 T minus 0 T. Avg = average. SD = Standard Deviation.*

Table S4 Maximum dose values for head and neck cancer patients treated with 6 x 6 Gy in mucosal structures

**Pt**	**Caudal 0-10 mm**			**Caudal 10-20 mm**			**Caudal 20-30 mm**			**Caudal 30-40 mm**			**Caudal 40-50 mm**			**Caudal 50-60 mm**		
	1.5 T	0 T	Dif	1.5 T	0 T	Dif	1.5 T	0 T	Dif	1.5 T	0 T	Dif	1.5 T	0 T	Dif	1.5 T	0 T	Dif
1	1.5	1.1	0.4	0.9	0.6	0.3	0.7	0.5	0.3	0.5	0.4	0.1	0.4	0.3	0.1	0.3	0.2	0.1
2	5.1	4.0	1.1	1.7	1.2	0.5	1.1	0.6	0.5	0.7	0.5	0.2	0.5	0.4	0.2	0.5	0.3	0.1
3	1.9	1.9	0.0	0.7	0.7	0.0	0.5	0.5	0.0	0.4	0.3	0.1	0.4	0.3	0.1	0.4	0.3	0.1
4	4.9	2.0	3.0	1.7	1.4	0.4	1.3	1.1	0.2	1.0	0.9	0.2	0.9	0.7	0.2	0.6	0.6	0.0
5	3.8	2.5	1.3	2.1	1.6	0.5	1.3	1.1	0.2	0.9	0.9	0.0	0.8	0.7	0.0	0.6	0.6	0.0
Avg	3.4	2.3	1.1	1.4	1.1	0.3	1.0	0.8	0.2	0.7	0.6	0.1	0.6	0.5	0.1	0.5	0.4	0.1
SD	1.5	1.0	1.0	0.5	0.4	0.2	0.3	0.3	0.2	0.2	0.2	0.1	0.2	0.2	0.1	0.1	0.2	0.0
	**Cranial 0-10 mm**			**Cranial 10-20 mm**			**Cranial 20-30 mm**			**Cranial 30-40 mm**			**Cranial 40-50 mm**			**Cranial 50-60 mm**		
	1.5 T	0 T	Dif	1.5 T	0 T	Dif	1.5 T	0 T	Dif	1.5 T	0 T	Dif	1.5 T	0 T	Dif	1.5 T	0 T	Dif
1	5.1	3.8	1.4	1.7	1.1	0.6	0.8	0.6	0.1	0.8	0.4	0.4	0.4	0.3	0.1			
2	18.0	9.3	8.7	3.5	1.3	2.1	1.2	0.9	0.3	0.8	0.7	0.2	0.7	0.4	0.4			
3	9.3	6.5	2.8	2.5	2.3	0.2	3.6	1.3	2.2	1.0	1.0	0.1	0.6	0.7	-0.2			
4	9.3	13.5	-4.2	5.4	3.2	2.3	3.5	2.2	1.4	1.9	1.2	0.7	1.6	1.0	0.5	0.9	0.9	0.0
5	7.0	4.9	2.1	2.2	1.8	0.4	2.2	1.4	0.8	1.1	1.0	0.1	0.8	0.7	0.1	0.6	0.5	0.1
Avg	9.7	7.6	2.2	3.1	1.9	1.1	2.2	1.3	1.0	1.1	0.9	0.3	0.8	0.6	0.2	0.7	0.7	0.1
SD	4.4	3.5	4.1	1.3	0.7	0.9	1.2	0.5	0.8	0.4	0.3	0.2	0.4	0.3	0.2	0.2	0.2	0.0

**All values are presented in Gray (Gy). Pt=patient. Dif = difference 1.5 T minus 0 T. Avg = average. SD = Standard Deviation.*

Figure S5

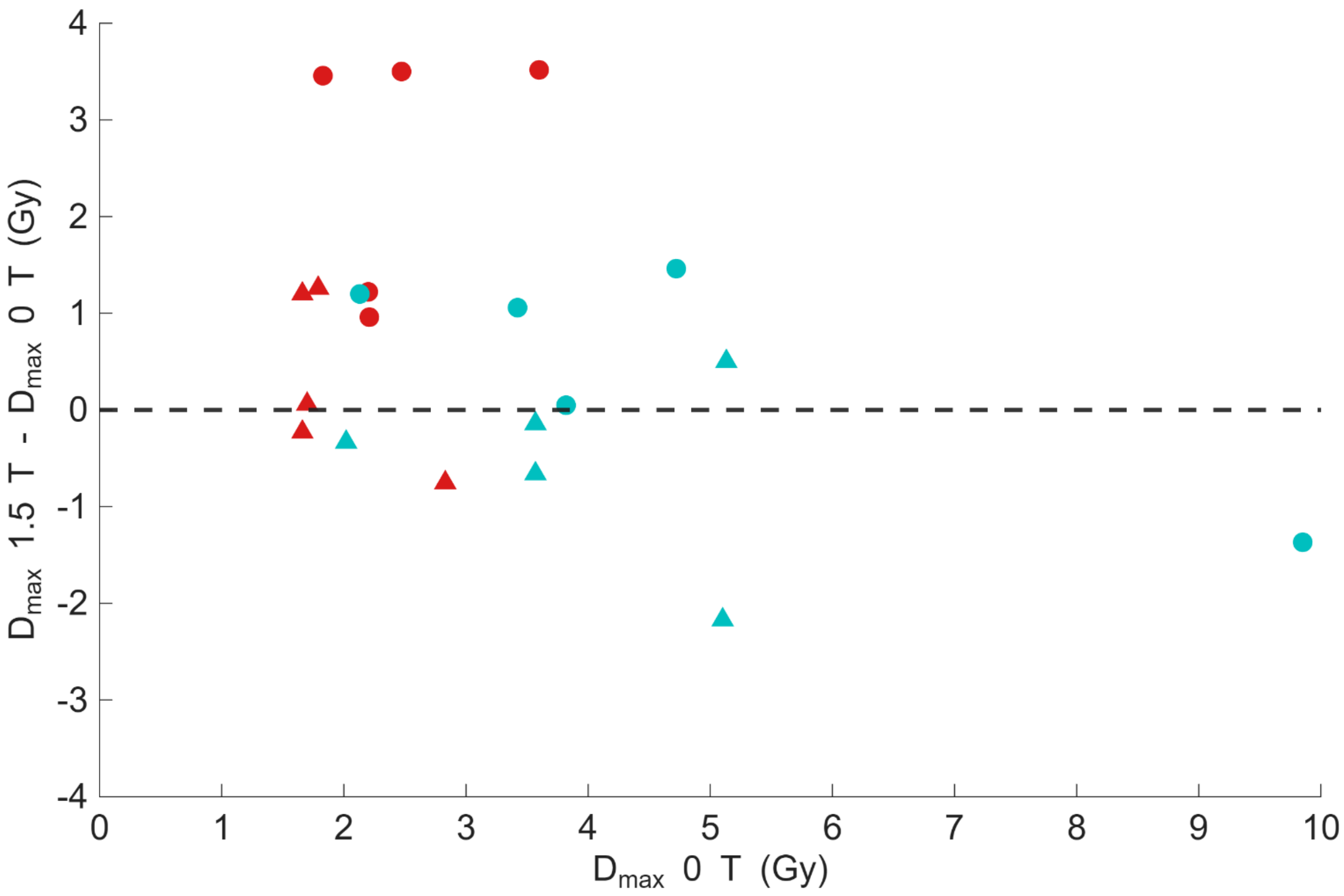

Quantification of the maximum dose in the 5 mm thick skin caudal to the high dose region (red) and cranial to the high dose region (blue), for head and neck cancer patients treated with a palliative regimen of 6 x 6 Gy (circles) and early-stage glottic cancer patients treated with 5 x 8.5 Gy (triangles).
